# Gene flow and adaptive potential in a generalist ectoparasite

**DOI:** 10.1186/s12862-018-1205-2

**Published:** 2018-06-19

**Authors:** Anaïs S. C. Appelgren, Verena Saladin, Heinz Richner, Blandine Doligez, Karen D. McCoy

**Affiliations:** 1 Evolutionary Ecology Laboratory, Institute of Ecology and Evolution, University of Bern, Baltzerstrasse 6, Bern, Switzerland; 2 CNRS; Université de Lyon, F-69000, Lyon; Université Lyon 1; Department of Biometry and Evolutionary Biology, LBBE UMR 5558, Bâtiment Gregor Mendel, 43 boulevard du 11 novembre 1918, F-69622, Villeurbanne, France; 3 Department of Biometry and Evolutionary Biology, LBBE UMR 5558, Bâtiment Gregor Mendel, Université Lyon 1, 43 boulevard du 11 novembre 1918, F-69622 Villeurbanne, France; 4 Maladies Infectieuses & Vecteurs: Ecologie, Génétique, Evolution & Contrôle (MIVEGEC), Université de Montpellier – CNRS – IRD, Centre IRD, 911 avenue, Agropolis, BP 64501, F-34000 Montpellier, France; 5 Department of Ecology and Genetics/Animal Ecology, Evolutionary Biology Centre (EBC), University of Uppsala, Norbyvägen 18D, 752 36 Uppsala, Sweden

**Keywords:** Multi-host system, Habitat fragmentation, Dispersal, Local adaptation, Ecological specialization, Spatial scale, Population genetics, *Ficedula albicollis*, *Parus major*, *Ceratophyllus gallinae*

## Abstract

**Background:**

In host-parasite systems, relative dispersal rates condition genetic novelty within populations and thus their adaptive potential. Knowledge of host and parasite dispersal rates can therefore help us to understand current interaction patterns in wild populations and why these patterns shift over time and space. For generalist parasites however, estimates of dispersal rates depend on both host range and the considered spatial scale. Here, we assess the relative contribution of these factors by studying the population genetic structure of a common avian ectoparasite, the hen flea *Ceratophyllus gallinae*, exploiting two hosts that are sympatric in our study population, the great tit *Parus major* and the collared flycatcher *Ficedula albicollis*. Previous experimental studies have indicated that the hen flea is both locally maladapted to great tit populations and composed of subpopulations specialized on the two host species, suggesting limited parasite dispersal in space and among hosts, and a potential interaction between these two structuring factors.

**Results:**

*C. gallinae* fleas were sampled from old nests of the two passerine species in three replicate wood patches and were genotyped at microsatellite markers to assess population genetic structure at different scales (among individuals within a nest, among nests and between host species within a patch and among patches). As expected, significant structure was found at all spatial scales and between host species, supporting the hypothesis of limited dispersal in this parasite. Clustering analyses and estimates of relatedness further suggested that inbreeding regularly occurs within nests. Patterns of isolation by distance within wood patches indicated that flea dispersal likely occurs in a stepwise manner among neighboring nests. From these data, we estimated that gene flow in the hen flea is approximately half that previously described for its great tit hosts.

**Conclusion:**

Our results fall in line with predictions based on observed patterns of adaptation in this host-parasite system, suggesting that parasite dispersal is limited and impacts its adaptive potential with respect to its hosts. More generally, this study sheds light on the complex interaction between parasite gene flow, local adaptation and host specialization within a single host-parasite system.

**Electronic supplementary material:**

The online version of this article (10.1186/s12862-018-1205-2) contains supplementary material, which is available to authorized users.

## Background

Long-term interactions between hosts and parasites can shape the evolution of their life history traits, their behavior and their physiology, and can alter the way they interact with other organisms in the environment [1]. Because parasites often show higher reproductive rates than their hosts, they are frequently considered to have a higher evolutionary potential and therefore an advantage in the evolutionary arms race [2, 3]. However, this assumption is not always verified, because parasites often have low independent dispersal capacities and can be subjected to strong population bottlenecks [4] reducing their adaptive potential by lowering genetic diversity within populations. In this sense, relative host-parasite dispersal rates among populations should be a key determinant of relative genetic diversity, and thus adaptive potential, and therefore should condition coevolutionary outcomes [2]. Indeed, evolutionary models predict that the more dispersive species should benefit from genetic novelty at the local scale, and therefore lead in the arms race [2, 3, 5], so long as dispersal is random with respect to genotype and does not completely homogenize populations [6].

The host range of a parasite is also an important determinant of coevolutionary outcomes. Indeed, a parasite which infests a broad range of hosts will be subject to diffuse selective pressures from each host type compared to a more specialist parasite, resulting in a lower probability of fixing beneficial alleles for generalists compared to specialists [5, 7, 8]. Host range can also influence parasite dispersal by altering parasite habitat range and host-linked dispersal probabilities. Host range can therefore influence host-parasite coevolution directly through selection, and indirectly through its consequences on dispersal. However, the definition of a parasite’s host range strongly depends on the spatial scale considered. Indeed, parasite species considered as generalists at the scale of their overall distribution can sometimes be composed of distinct local populations specialized on different hosts [9–14]. Population-based studies are therefore essential for characterizing host range and spatial population structure at fine spatial scales [15]. Although a strong link is to be expected between host specialization and local adaptation, studies focusing on biological systems experiencing these two phenomena at the same time have received little attention.

The present study aimed at characterizing the population structure of a “generalist” ectoparasite at different spatial scales, and among different host species, in order to examine the link between dispersal, patterns of local adaptation and host specialization. We focused on a common bird ectoparasite, the hen flea *Ceratophyllus gallinae*, infesting hole-nesting passerine species [16]. This nest-based parasite can negatively impact the reproductive success of its host by decreasing nestling survival and growth and by increasing the costs of reproduction [17–19]. Members of the Paridae family are considered as the main hosts for this parasite based on prevalence and intensity records, but other families are also commonly infested, in particular the Muscicapidae family [16]. An experimental test of local adaptation of hen fleas to great tit hosts (*Parus major,* Paridae) in a fragmented habitat composed of distinct wood patches on Gotland (Sweden), suggested that hen fleas are locally maladapted to their hosts, i.e. local fleas have higher fitness when exploiting non-local compared to local tit hosts [20]. This observation could be explained by lower relative dispersal of fleas among patches compared to great tits. A reciprocal transfer of hen fleas between nests of great tits and an alternative host, the collared flycatcher (*Ficedula albicollis,* Muscicapidae), also suggested the presence of distinct flea populations associated with each host species in some localities [21]. This observation could be due to reduced flea gene flow between host species within the same patch and/or strong selection for host adaptation. Here, we used a population genetic approach to characterize flea population structure over space and host species, and thereby assess whether the previously observed patterns (local maladaptation and local host specialization of fleas) are associated with expected patterns of relative host-parasite gene flow.

## Methods

### Sampling

**Fig. 1:**
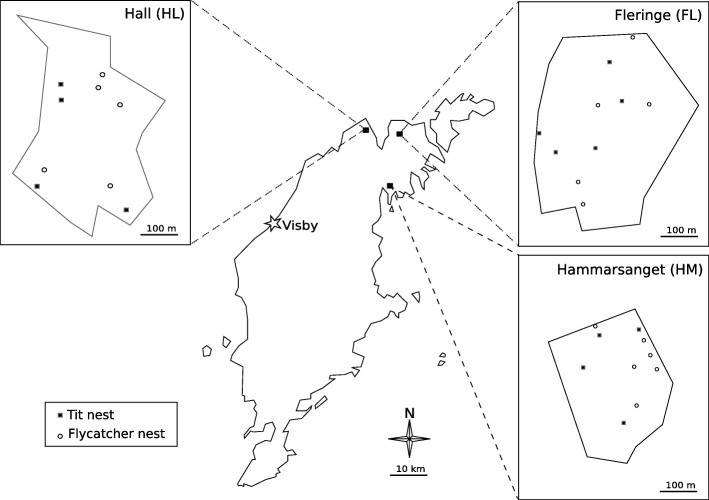
Location of the sampling sites on Gotland

In March 2013, we sampled old nests of great tits and collared flycatchers in three wood patches (Fleringe (FL), Hall (HL) and Hammarsänget (HM)) on the northern part of the Swedish island of Gotland (Fig. 1). Wooden nest boxes were erected in these patches in 2004. From 2004 to 2007, great tit and flycatcher nests were monitored in these patches without manipulating flea populations, except by the removal of old nests from nest boxes at the end of the season. Starting in 2007, these patches were no longer monitored, allowing fleas to establish natural population dynamics prior to our sampling. Old nests were collected in separate hermetic plastic bags and sorted by bird species based on the material used to build the nest. Collared flycatcher nests are mainly composed of dry grasses and leaves, whereas great tits nests contain moss and fur. Some risk of confusion between nests of great tits and blue tits (*Parus caeruleus*), a closely related species with similar ecology, was possible in our sample. As blue tits are about half as abundant as great tits in our patches and frequently use feathers as nest material in addition to fur, few blue tit nests were likely included among the sampled nests. However, to remain conservative, we only make the distinction between “flycatcher nests” (collared flycatchers) and “tit” nests in our study.

Live adult fleas were collected from each sampled nest using an insect aspirator and were stored in 95% alcohol. A total of 67 nests were collected in the three patches, with an overall prevalence of hen fleas of 47% in tit nests, and 49% in flycatcher nests. Among the 35 sampled nests with a high enough number of fleas (> 20), 29 nests were selected at random for genotyping, with a balance between patches and host species (for Hall: 4 tit and 5 flycatcher nests, for Fleringe: 5 tit and 5 flycatcher nests, for Hammarsänget: 4 tit and 6 flycatcher nests). We genotyped 19 to 22 fleas from each selected nest. Fleas sampled within a nest are considered as an infrapopulation.

**Fig. 2:**
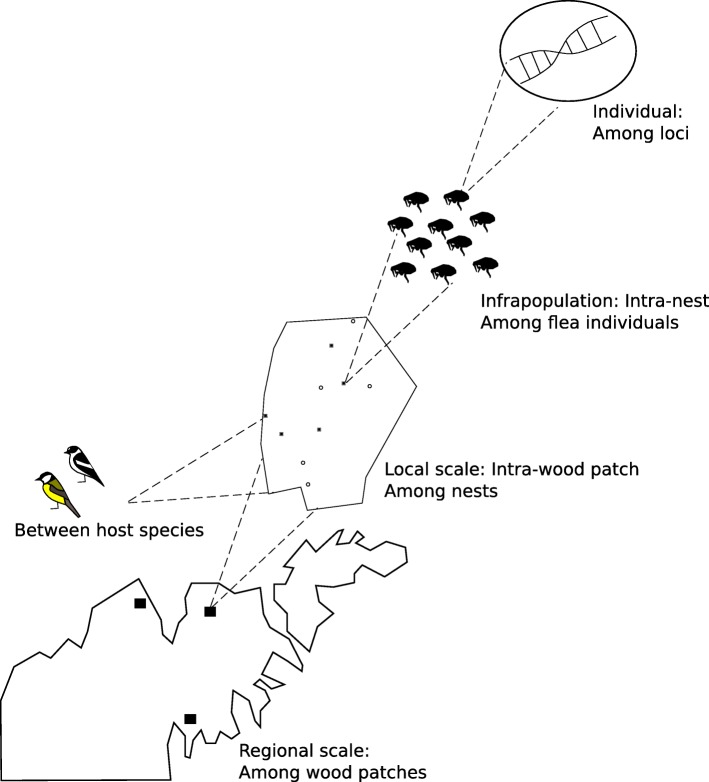
Hierarchical levels of the study design

Maps summarizing the sampling locations and methods (Figs. 1 and 2) were produced using the vector drawing software Inkscape 0.91.

### Deoxyribonucleic acid extraction, microsatellite markers and genotyping

We individually extracted DNA from fleas in 300 μL of an extraction mix prepared with 234 μL of Nuclei Lysis Solution (Promega AG, Dübendorf, Switzerland), 56 μL of 0.5 M Ethylenediaminetetraacetic acid (EDTA; Fluka, Sigma-Aldrich, Buchs, Switzerland) and 10 μL of Proteinase K (Promega AG, Dübendorf, Switzerland). We then performed DNA purification using magnetic beads (MagneSil Blue, Promega AG, Dübendorf, Switzerland), following the manufacturer’s protocol.

Polymerase Chain Reactions were performed in a 10 μL volume, containing 2 μL of the DNA extract, 5 μL of QIAGEN Multiplex PCR Master Mix (QIAGEN AG, Hombrechtikon, Switzerland), 1 μL of fluorescent primer mix (2 to 6 μM), and 2 μL of molecular grade water. DNA amplifications were carried out using a Geneamp 9700 Thermocycler (Applied Biosystems, Rotkreuz, Switzerland). PCR conditions were the same for all targeted markers: initial denaturation at 95 °C for 15 min, followed by 35 cycles of 30s at 94 °C for denaturation, 90s at 57 °C for primer annealing and 60s at 72 °C for elongation and ending with a final elongation step of 72 °C for 10 min.

We targeted 13 of the 23 markers previously described by Binz et al. [22] for *C. gallinae* genotyping, focusing on the most reliable (i.e. good amplification success, low null alleles): Cga2, Cga3As, Cga6, Cga9, Cga11, Cga14, Cga26, Cga28, Cga31, Cga32, Cga42, Cga45 and Cga46. We assessed allele lengths using the genetic analyzer ABI 3100 (Applied Biosystems) and the software Genemapper v3.7 (Applied Biosystems).

### Assessment of marker quality

In order to verify marker independence, we tested for linkage disequilibrium among the 13 markers over all infrapopulations using the software Genepop v. 4.3 [23] with default values for dememorization, batches and iteration numbers. We assessed for the occurrence of null alleles, stuttering and allele dropout for each locus using the software Micro-Checker v.2.2.3 [24]. Genetic diversity and allelic richness were assessed with the software FSTAT v.2.9.3.2. [25]. To determine whether markers conformed to proportions expected under Hardy-Weinberg equilibrium, and to assess if all markers gave coherent information, i.e. conformed to neutrality, we also quantified F_is_ and F_st_ for each locus over all populations using Weir and Cockerham’s unbiased estimator f and θ, respectively [26], and calculated the standard error of these estimates by jackknifing over infrapopulations using FSTAT v.2.9.3.2.

### Analysis of genetic structure at different spatial scales and between host species

We tested for flea population genetic differentiation within and among wood patches and investigated the relative genetic structure at the different spatial levels of the system (Fig. 2).

#### Among patches

We characterized F_st_ at the among patch level using Weir and Cockerham’s unbiased estimator θ [26] and calculated its standard error by jacknifing over patches using FSTAT v.2.9.3.2. Significance was assessed using 10,000 permutations. Because most loci showed high polymorphism, the maximum estimate of differentiation is lower than 1. We therefore also calculated the F_st max_ as suggested by Hedrick and Goodnight [27], using the programs RecodeData [28] and FSTAT v.2.9.3.2. Standardized differentiation was then calculated as F_st standardized_ = F_st obs_ / F_st max_ [28]. Population structure at the among patch scale was also graphically explored using a DAPC (Discriminant Analysis of Principal Components) implemented in the package adegenet 1.4–2 [29] for the software R v.3.0.2 [30]. This analysis combines an initial principal component analysis (PCA, considering among-individual variation) with a discriminant analysis (DA, considering among-group variation). Based on cumulative variance and eigenvalues, we retained 100 principal components for the PCA, and two discriminant functions for the DA. Finally, the relative contribution of different hierarchical spatial scales (from the among patch scale to the intra-individual level, Fig. 2) in explaining observed genetic variation was investigated using an analysis of molecular variance (AMOVA) computed by GenAlEx v.6.5 [31, 32] with 9999 permutations.

#### Within patches and between host species

We examined the population genetic structure of fleas within each of the three patches, both among nests and between host species. First, for each patch, we calculated F_st_ and F_st max_ among nests as described above. Second, we tested for genetic differentiation between hosts in each patch using AMOVAs to decompose molecular variance from the host species category to the intra-individual level. Third, we investigated clustering among nests within each patch using DAPC as described above (with 50 components and 2 discriminant functions). Fourth, in order to test if the nest level was the most relevant minimal hierarchical scale for our study and to identify potential genetic groups shared by different nests, we also carried out a clustering analysis for each patch using the software STRUCTURE v.2.3.4 [33, 34]. We set the possible number of clusters (k) from 1 to 20 (assuming a maximum of two subpopulations within each nest), the burn-in period to 500,000 iterations, and the number of Markov chain Monte Carlo iterations to 1,000,000. Prior information about infrapopulation identity was included in the analysis. The optimal number of clusters (k) for each patch was assessed with STRUCTURE HARVESTER v 0.6.94 [35] using the Evanno’s Delta k value [36]. Fifth, in order to explore whether gene flow occurred among neighboring nest boxes, we tested for isolation by distance using a linearized estimate of genetic distance (F_st_/(1-F_st_)) and the natural logarithm of the geographic distance between nests (in meters) using Mantel tests (10,000 permutations; program Genepop v. 4.3). Where host-associated differentiation among flea populations was found, this test was carried out separately for each host species. Finally, we used Teriokhin’s generalized binomial procedure implemented in the software Multitest v1.2 [37, 38] to combine independent tests. As there were only 3 tests to combine, the entire set of *p*-values was used rather than just half [39]. For three independent tests, the optimal threshold value of significance is < 0.3689.

#### Within nests

In this last analytical step, we examined the population genetic structure at the infrapopulation level (within nests). We tested for a departure from panmixia within nests by calculating the F_is_ (f) for each patch with FSTAT v.2.9.3.2. We also calculated mean relatedness within each nest with Queller and Goodnight’s coefficient [40] using GenAlEx v.6.5. The standard error and significance of the estimators were assessed based on 9999 permutations. We also used the results of the previous clustering analysis at the patch level (performed with STRUCTURE v.2.3.4) to assess whether sub-structure occurred within infrapopulations.

## Results

### Assessment of marker quality

Significant linkage disequilibrium was observed between two marker pairs: Cga31 and Cga46 (one nest in Fleringe), and Cga31 and Cga32 (one nest in Fleringe, two in Hall and one in Hammarsänget). This result could be due in theory to consanguinity within some nests, but this explanation was not supported by the observed values of F_is_ and relatedness within infrapopulations (see within-nest section) and suggests that the markers may be physically linked. We therefore removed marker Cga31 from the marker set.

**Fig. 3:**
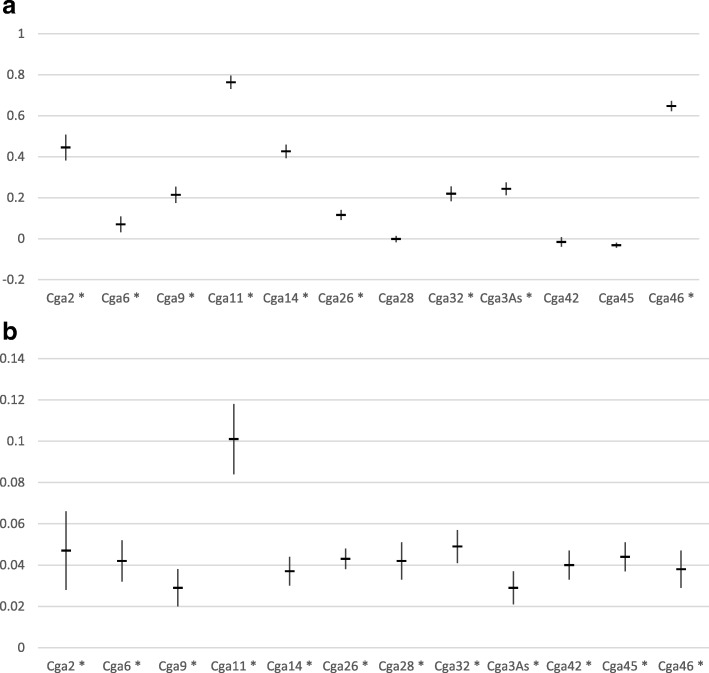
**a** F_is_ (f ± SE) and (B) F_st_ (θ ± SE) for each locus, over all infrapopulations. A star (*) next to the locus label indicates cases where *p* < 0.05, all markers gave a significant estimate in (**b**)

Genetic diversity and allelic richness were variable among markers, ranging from 0.42 to 0.88 on average for genetic diversity, and from 5 to 32 over all populations for allelic richness, but did not vary much among nests or patches (see Tables S1 and S2 in Additional file 1). F_is_ estimates were also variable among markers, but most estimates were significantly different from zero (Fig. 3a). In contrast, all markers gave similar estimates of F_st_ (Fig. 3b), except for the marker Cga11, which tended to overestimate differentiation. Markers Cga 11, Cga 46, Cga3As and Cga14 showed some evidence for null alleles, which may have altered F_is_ estimates at these markers. No allele dropout or stuttering was suggested based on patterns of allele frequencies and allele sizes. To maintain reasonable power, the whole set of 12 markers was retained for subsequent analyses, but the potential influence of null alleles was controlled by running every analysis again without the four markers concerned (i.e. on a set of 8 markers). Results did not differ with or without these markers, except for the estimation of global F_is_, which was lower with the set of 8 compared to 12 markers, as expected. However, the infrapopulation estimates of F_is_ remained significantly higher than zero even with the reduced marker set (for 12 markers: f ± SE for 12 and 8 markers respectively: 0.239 ± 0.074 and 0.104 ± 0.046, *p* = 0.0001 in both cases). Deviations from Hardy-Weinberg proportions therefore originated from biological factors rather than technical issues (see below).

### Among patches

**Fig. 4:**
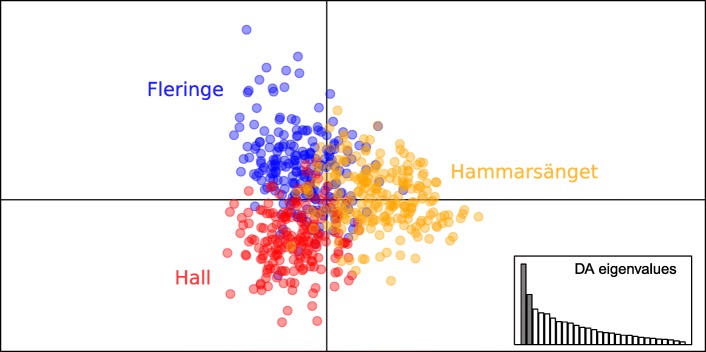
Discriminant analysis of principal components representing among-nest genetic structure at the among patch scale. Proportion of among group variation is 47.0% on the horizontal and 29.7% on the vertical axis

Overall structure considering nests from all patches was significantly greater than zero (F_st_ (θ) ± SE =0.044 ± 0.006, p = 0.0001). Given that the maximum F_st max_ value calculable with this dataset is 0.265 instead of 1, the actual value of differentiation is F_st standardized_ = 0.166. At this large scale, differentiation was mostly driven by spatial components, and the DAPC separated infrapopulations into three groups corresponding to the three patches (Fig. 4). However, patches do not appear totally differentiated, as the three groups overlap on the two main axes. AMOVA analyses supported these findings. Although most molecular variation was attributed to the intra-individual (inter-loci) and intra-nest (inter-individuals) levels, the inter-nest and inter- patch levels explained low, but significant proportions of the variation, i.e. 3 and 1.3% respectively (Table 1).

**Table 1:** Decomposition of genetic variation from among patch to intra-individual scales, ignoring host-associated structure

Spatial level	Df	% variation	Statistics	pvalue
Among wood patches	2	1.3	Frt = 0.013	< 0.0001
Among nests/ Within wood patches	26	3.0	Fsr = 0.030	< 0.0001
Among individuals/ Within nests	598	26.8	Fst = 0.042	< 0.0001
Within individual	627	69.0	Fis = 0.280	< 0.0001

### Within patches

Within each wood patch, overall among-nest differentiation was significantly differently from zero (F_st_ (θ) ± standard error: Fleringe: 0.035 ± 0.006, Hall: 0.021 ± 0.004, Hammarsänget: 0.044 ± 0.006; all *p*-values< 0.0001). Once standardized for polymorphism, these values of F_st_ were F_st standardized_ = 0.138, 0.085 and 0.166 for Fleringe, Hall and Hammarsänget, respectively.

**Fig. 5:**
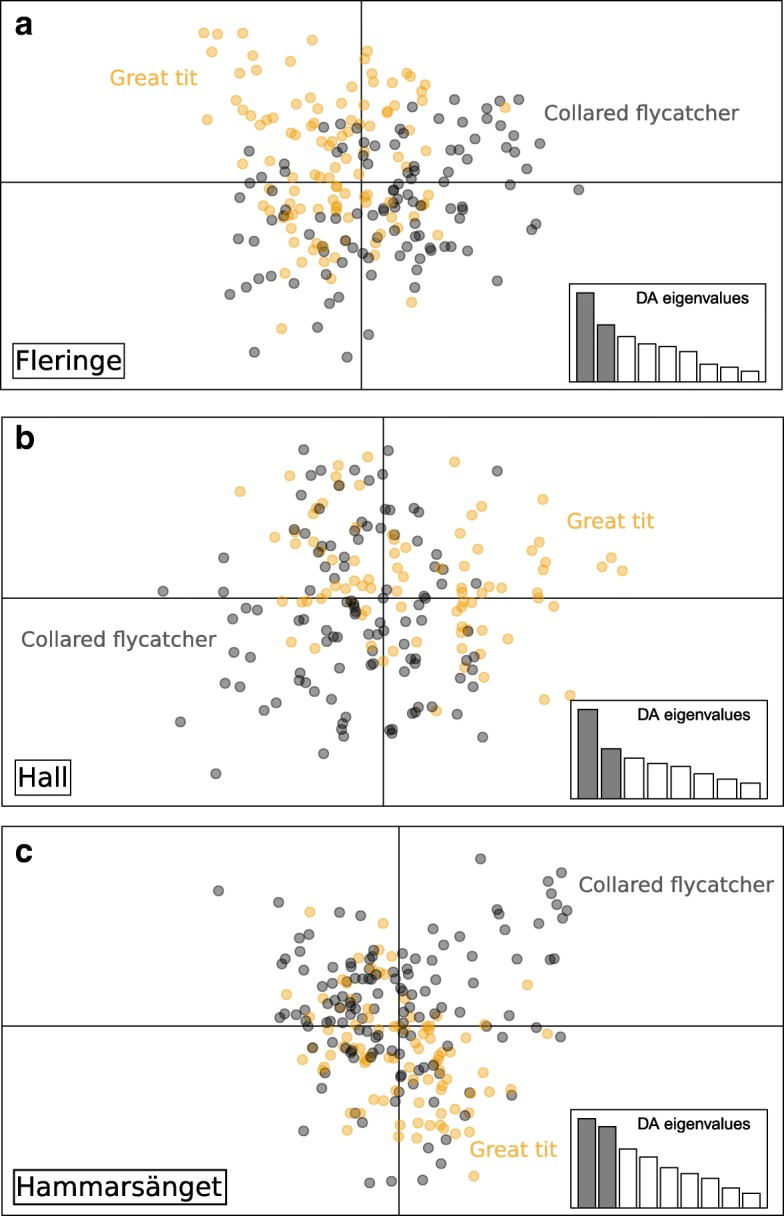
Discriminant analysis of principal components representing among-nest genetic structure within patches. **a** Fleringe: Proportion of between group variation is 41.2% on the horizontal and 26.1% on the vertical axis. **b** Hall: Proportion of between group variation is 35.5% on the horizontal and 20.4% on the vertical axis. **c** Hammarsänget: Proportion of between group variation is 38.6% on the horizontal and 34.8% on the vertical axis

The DAPC analysis revealed no obvious pattern of population structure in relation to host type (Fig. 5). The decomposition of total genetic variation within each patch revealed low but significant differentiation between host-associated flea populations in two of the three patches at a threshold of 5% (Fleringe and Hammarsänget, but not in Hall: Table 2). However, based on the threshold value expected for k = 3 tests calculated by the generalized binomial procedure (*P* ≤ 0.3689), the overall pattern of between-host differentiation was significant.

**Table 2:** Decomposition of genetic variation from the between-host to the intra-individual scales within each patch

Wood patch	Fleringe				Hall				Hammarsänget			
	Df	%var	Stats	*P*value	Df	%var	Stats	*P*value	Df	%var	Stats	*P*value
Between hosts	1	0.4	Frt = 0.004	0.002	1	0.2	Frt = 0.002	0.092	1	0.2	Frt = 0.002	0.020
Among nests / Within hosts	8	2.8	Fsr = 0.028	< 0.0001	7	1.7	Fsr = 0.017	< 0.0001	8	3.9	Fsr = 0.039	< 0.0001
Among individuals / Within nests	205	27.7	Fst = 0.032	< 0.0001	184	27.1	Fst = 0.019	< 0.0001	209	26.6	Fst = 0.041	< 0.0001
Within individuals	215	69.2	Fis = 0.286	< 0.0001	193	71.0	Fis = 0.276	< 0.0001	219	69.3	Fis = 0.277	< 0.0001

Evanno’s Delta k method suggested an optimal number of clusters of k = 17, 2 and 5 for Fleringe, Hall and Hammarsänget respectively. We also compared the patterns of assignment to clusters corresponding to the second highest Delta k value (Fleringe: k = 15; Hall: k = 10; Hammarsänget: k = 9) to test how robust the genetic structure was with respect to the number of assumed clusters. Regardless of the k-value considered, the results of STRUCTURE indicated shared group memberships among nests and no obvious correspondence with host species (see Additional file 2 for graphical results).

The test for isolation by distance was significant for Hall (*p* = 0.01), where between host differentiation was not significant at the 0.05 threshold. In the two other patches, isolation by distance signals differed between host species, (Fleringe: overall: *p* = 0.07, among tit nests: *p* = 0.105, among flycatcher nests: *p* = 0.025; Hammarsänget: overall: *p* = 0.07, among tit nests: *p* = 0.040, among flycatcher nests: 0.740; see Additional file 3 for graphical results). The *p*-values obtained corresponded to an overall significant pattern of isolation by distance across patches according to the generalized binomial procedure.

### Within nests

**Fig. 6:**
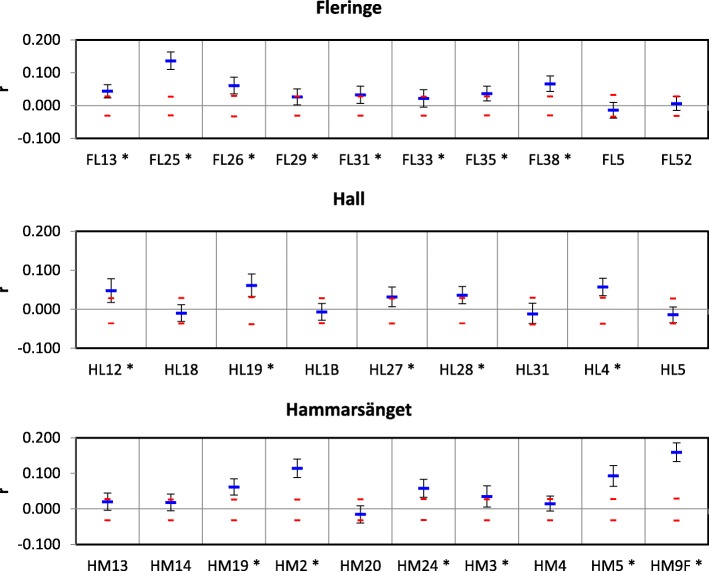
Per infrapopulation average relatedness (blue lines) ± SE (black lines). Red lines show the upper and lower bounds of expected values under a random hypothesis. A star (*) next to the nest label indicates cases where *p* < 0.05

Patch-wide average F_is_ estimates per nest were all significantly greater than zero (F_is_ values: Fleringe: 0.246 ± 0.078, Hall: 0.242 ± 0.075, Hammarsänget: 0.232 ± 0.071, all *p*-values< 0.0001) suggesting within nest substructure and/or inbreeding. For all three patches, mean pairwise relatedness within each nest indicated that individuals within a nest were frequently more closely related than individuals selected at random in the patch (relatedness significantly higher than random: 8/10 nests in Fleringe, 5/9 in Hall and 6/10 in Hammarsänget; Fig. 6). Moreover, clustering analyses suggested that some nests could shelter different flea sub-populations (See for instance nests HM19, HM9 and HL 19 in Additional file 2), corresponding to the co-existence of different flea lineages within nests.

## Discussion

In this study, we assessed the population genetic structure of a common avian ectoparasite exploiting two of its main host species at different spatial scales in a fragmented landscape, i.e. within and among populations and infrapopulations. Our study aimed to test whether this genetic structure matched expected patterns based on previous experimental results that revealed parasite maladaptation and host specialization in part of the study area [20, 21]. Based on these patterns, we hypothesized that flea population structure should reveal low among-nest and/or among-patch gene flow and signs of host-associated genetic divergence, at least in some localities. Our results show significant genetic structure occurs at all spatial scales investigated and between hosts, which suggests low overall dispersal of hen fleas.

### Differentiation of hen fleas among nests and patches

As genetic variation was significantly structured at the level of the wood patch, the three woods sampled here were treated as three replicates for studying fine-scale spatial structure. Within each patch, nests were genetically differentiated, with standardized values of F_st_ ranging from 8 to 17%. The low overall dispersal of hen fleas we found contradicts previous observations in which artificially deparasitized nest boxes were rapidly recolonized due, presumably, to high dispersal rates [41]. However, these observations could be due to fleas of the same infrapopulations remaining outside, but close to boxes and not to among-box dispersal per se. Moreover, not all dispersal events may be followed by successful reproduction and therefore lead to gene flow. Indeed, density-dependent reproduction has been described in hen flea infrapopulations [42] and may prevent dispersers from achieving reproductive success when they arrive in already abundant infrapopulations.

Given among patch differentiation and the tendency for significant patterns of isolation by distance among nests within patches, hen flea dispersal is likely to be a step by step process, with fleas dispersing over short distances between neighboring nests. Fleas are thought to disperse mostly at the beginning of spring, when adults emerge from overwintering cocoons in response to increasing temperatures [43, 44]. Fleas can disperse either by jumping/ crawling on the ground [43] or by jumping on prospecting birds when they visit cavities [44]. Dispersal may also be possible at the end of the breeding season with the post-breeding prospecting movements of fledglings or adults [45] or with accidental hosts such as small mammals that temporarily use nest boxes. However, as passerines and small rodents exhibit preening/grooming behaviors [46], such a dispersal mechanism is more likely to occur over short distances. Laboratory experiments are now called for to quantify the potential for independent flea dispersal from one cavity to another, and therefore evaluate the relative use of independent vs host-associated dispersal.

Among patch differentiation in fleas that takes into account both inter- and intra-nest variation (F_rt_ = 0.013) is approximately twice as high as that previously estimated for great tits on Gotland at the among patch level (for 10 patches with distances ranging from 3 to 50 km, F_st_ = 0.006, [47]). Although this estimate for great tits was not standardized for the F_stmax_ value [27], the levels of polymorphism observed for great tits [47, 48] and hen fleas (here) are similar and allow us to make a direct comparison of the two raw values. Hen flea maladaptation on Gotland, revealed experimentally [20], could therefore be at least partly explained by the lower relative dispersal of the parasite compared to its main host [2]. Although intuitive, lower parasite dispersal compared to their hosts may not be a general trend. A recent meta-analysis showed that parasites are frequently less structured than their hosts [49]. As an example, higher parasite dispersal was inferred in a system composed of bats and wingless bat flies [50]. Bat flies typically live in the host fur or on wing membranes [50, 51], which could result in a rather frequent bat to bat dispersal. In contrast, hen fleas live in nest material rather than on the host itself [42] reducing this dispersal potential. Because hen fleas live largely off-host, their reproductive success also depends on local environmental conditions [42]; in bat flies incubate larvae within their abdomen [51] and reproductive success after dispersal may thus be less dependent on new local conditions. Finally, bats are colonial animals [52] which can favour effective parasite transmission, compared to more solitary breeders like great tits and collared flycatchers. In general, therefore, the dispersal ability of ectoparasites, and their associated population genetic structure, will depend on a combination of parasite and host biology, ecology and social behaviors.

### Population structure of hen fleas within nests

We found that flea infrapopulations (i.e., fleas within a nest) deviated significantly from Hardy-Weinberg proportions. This result may be due to either inbreeding (i.e. mating with relatives) and/or the presence of different family groups within nests (i.e.*,* a local Wahlund effect, [53]); both processes likely occurred within nest boxes. Inbreeding is supported by the fact that most nests showed higher relatedness values than expected in the overall population of each patch. A Wahlund effect is supported by clustering analyses, which indicated the co-existence of fleas from different clusters within some nests. Clusters may be due either to the presence of dispersing fleas that did not yet mix with the rest of the local nest population, or to a particular mating structure within nests such as homogamy (i.e. mating with individuals with similar traits, such as size [53]). Given the relatively low dispersal rate suggested here for the flea, this latter explanation may be more likely. Moreover, multiple mating has been described in this flea species [54], which should enhance the rapid genetic mixing of local individuals and dispersers if mating is random.

### Between host differentiation of hen fleas

Population differentiation in relation to host species was observed here, suggesting that despite small scale gene flow, population divergence is occurring between fleas exploiting tits and flycatchers. This result matches fitness differences observed in a cross-infestation experiment of hen fleas between great tits and collared flycatchers on the same island [21]. In this experiment: (i) fleas originating from tit nests tended to cause higher damage to tit hosts compared to fleas originating from flycatcher nests, and (ii) fleas originating from flycatcher nests frequently had faster larval development rates than fleas originating from tit nests when infesting flycatcher nests. However, in this experiment, the effect of flea origin differed among localities. This spatial variation in apparent host specialization matches the variable pattern of population genetic structure among patches we found in the present study and suggests a geographic mosaic-like pattern in the coevolutionary interactions between fleas and their bird hosts [55]. Although the two host species considered here share the same breeding habitat and are present in similar abundances in the studied patches, they have contrasting life history and ecological traits that may exert divergent selection on hen fleas. First, collared flycatchers are trans-saharian migratory birds, whereas great tits are resident or partial, short-distance migrants. Migratory behavior can modify breeding phenology and alter energy allocation among functions, particularly to immune function [56–58]. Second, great tits and collared flycatchers use different nest materials. The moss used by great tits was suggested to modulate the development of fleas and alleviate parasitic costs for hosts [19]. Finally, great tits have longer reproductive periods and larger clutch sizes than collared flycatchers [59]. Great tits may therefore provide more food resources for adult fleas and a suitable environment for the development of flea larvae (regarding e.g. heat or humidity) for a longer period than flycatchers.

Because fleas sampled in the nest of a given host species were suggested to perform less well when infesting the alternative host [21], host specialization may act to reinforce population isolation. If offspring from crosses between fleas specialized on different host species perform poorly on both host species, for example, assortative mating by host species of origin could be favored and effective dispersal would be limited. Isolation among fleas infesting different host species could also result from great tits and collared flycatchers using slightly different microhabitats within patches due to interspecific competition for resources, as observed between pied and collared flycatchers in a sympatric zone [60]. Preferences for particular tree types or forest coverage could limit flea dispersal to alternative host nests. Flea dispersal could also be actively biased toward the host they are specialized on. This requires that fleas are able to discriminate among hosts during dispersal, a hypothesis that could be investigated in future experiments. Indeed, experimental work in a rodent-flea system showed that flea species used odor cues to discriminate between alternative sympatric host species [61]. Finally, great tits start breeding a couple of weeks earlier than collared flycatchers [59]. This difference could also lead to the temporal isolation of flea populations.

Hen flea populations on Gotland could either be at an equilibrium between selection and gene flow, such that the between-host genetic structure observed here is maintained across generations, or they could be undergoing specialization that could lead to increasing population structure between the different host species over time (i.e. ecological speciation with gene flow; [62]). The sampled populations in our study were only left undisturbed for a relatively short time before sampling (6 years) and may therefore be at the beginning of the specialization process. Repeat sampling of the same patches over several years using a non-destructive method (i.e. sampling fleas without destroying the nests) would be required to assess whether the observed between-host structure we found may lead to sympatric speciation or whether gene flow and/or inter-annual stochasticity may balance out host-associated patterns of divergence [4, 63]. Additionally, although difficult to obtain, fleas sampled from natural bird cavities would be useful to check whether results from artificial cavities correspond to interactions under natural breeding conditions.

## Conclusions

Hen flea populations were genetically structured across the study area and the observed patterns of spatial and host-associated differentiation fell in line with the results of previous experiments in this multi-host-parasite system. Our results suggest that the observed hen flea maladaptation to its main host, the great tit, could be explained by low parasite dispersal compared to its host. Limited dispersal may also account for the between-host patterns we found, where low gene flow favors host-associated selection and adaptation. The results of our population genetic study therefore provide essential new elements for understanding the ecology and coevolutionary trajectories of the hen-flea passerine system. Because this system represents a popular model system to study host-parasite interactions and evolution in the wild [1, 54, 64–67], our results are particularly relevant for other studies, especially experimental studies involving parasite translocations. For example, because infrapopulations within a single patch are genetically differentiated among and within host species, controlling for flea origin in experiments may be crucial. The generality of the presented results should now be tested in other study areas of this host-parasite system, and in particular in areas with different host communities.

More generally, our study sheds empirical light on the relationship between the two evolutionary phenomena acting here: local adaptation and host specialization. We suggest that there may be a feedback loop between local maladaptation and host specialization; low dispersal of the parasite can prevent local adaptation of the parasite population, but it can also prevent parasites from shifting hosts within their lifetime, therefore enhancing host-associated selective pressures on the parasite. Parasite specialization to particular host types may then, in turn, reduce the dispersal potential of the parasite (as dispersal to the wrong host type would result in low reproductive success) and enhance parasite maladaptation. Modelling and experimental studies with fast-evolving organisms would be helpful to test this relationship, and would provide exciting new perspectives for understanding the evolutionary ecology of host-parasite systems.

## Additional files

Additional file 1: This file contains two tables: **Table S1.** Genetic diversity at each locus for each population & **Table S2.** Allelic richness at each locus for each population (PDF 162 kb).

Additional file 2: This file contains figures representing the probability of individual flea assignment to the k clusters computed by STRUCTURE, for each patch (PDF 202 kb).

Additional file 3: This file contains graphs representing the isolation by distance within each patch, not considering host-associated structure (PDF 86 kb).

## Abbreviations

FL: Fleringe
HL: Hall
HM: Hammarsänget
DNA: Deoxyribonucleic acid
PCR: Polymerase Chain Reaction
EDTA: Ethylenediaminetetraacetic acid
DAPC: Discriminant Analysis of Principal Component
PCA: Principal Component Analysis
DA: Discriminant Analysis
AMOVA: Analysis of Molecular VAriance
