# Supplementary material for: Gene flow and adaptive potential in a generalist ectoparasite
Source: BMC Evol Biol. 2018 Jun 19;18:99. doi: 10.1186/s12862-018-1205-2 (PMC6009953; doi:10.1186/s12862-018-1205-2)
Supplement: Supplementary file 1 — This file contains two tables: Table S1. Genetic diversity at each locus for each population & Table S2. Allelic richness at each locus for each population (PDF 162 kb). [file 12862_2018_1205_MOESM1_ESM.pdf]

Additional file 1

Table S1: Genetic diversity at each locus for each population. Colors are attributed according to the value of genetic diversity (lower values in green, higher values in red)

Marker	FL13	FL25	FL26	FL29	FL31	FL33	FL35	FL38	FL5	FL52	HL12	HL18	HL19	HL1B	HL27
Cga2	0.38	0.36	0.52	0.42	0.61	0.49	0.29	0.46	0.48	0.54	0.40	0.45	0.30	0.34	0.40
Cga3as	0.73	0.62	0.62	0.69	0.73	0.69	0.72	0.59	0.74	0.75	0.76	0.69	0.66	0.72	0.72
Cga6	0.52	0.51	0.55	0.50	0.58	0.54	0.64	0.52	0.62	0.55	0.50	0.62	0.64	0.59	0.64
Cga9	0.82	0.61	0.62	0.70	0.77	0.76	0.75	0.71	0.82	0.72	0.70	0.81	0.79	0.81	0.83
Cga11	0.67	0.78	0.81	0.75	0.56	0.79	0.89	0.53	0.87	0.89	0.86	0.86	0.77	0.91	0.61
Cga14	0.94	0.84	0.93	0.84	0.81	0.89	0.94	0.86	0.87	0.90	0.87	0.91	0.95	0.87	0.79
Cga26	0.93	0.88	0.86	0.89	0.93	0.88	0.88	0.88	0.89	0.88	0.86	0.86	0.88	0.91	0.90
Cga28	0.82	0.80	0.80	0.86	0.82	0.88	0.87	0.92	0.89	0.89	0.79	0.86	0.77	0.90	0.87
Cga32	0.79	0.73	0.69	0.83	0.79	0.83	0.75	0.83	0.85	0.77	0.80	0.83	0.85	0.77	0.72
Cga42	0.66	0.67	0.73	0.69	0.69	0.71	0.71	0.64	0.70	0.73	0.58	0.71	0.59	0.74	0.68
Cga45	0.85	0.75	0.86	0.85	0.85	0.87	0.78	0.81	0.83	0.88	0.84	0.90	0.81	0.85	0.91
Cga46	0.79	0.77	0.87	0.90	0.84	0.88	0.90	0.84	0.90	0.87	0.80	0.85	0.74	0.91	0.80

Marker	HL28	HL31	HL4	HL5	HM13	HM14	HM19	HM2	HM20	HM24	HM3	HM4	HM5	HM9F
Cga2	0.32	0.52	0.18	0.54	0.34	0.42	0.28	0.60	0.41	0.28	0.43	0.54	0.50	0.32
Cga3as	0.74	0.73	0.76	0.79	0.83	0.74	0.64	0.79	0.59	0.74	0.68	0.75	0.68	0.75
Cga6	0.58	0.56	0.52	0.61	0.67	0.61	0.53	0.64	0.61	0.57	0.61	0.59	0.49	0.42
Cga9	0.74	0.86	0.79	0.76	0.74	0.71	0.84	0.79	0.71	0.70	0.75	0.70	0.70	0.74
Cga11	0.78	0.79	0.73	0.84	0.75	0.83	0.77	0.78	0.72	0.72	0.77	0.76	0.57	0.68
Cga14	0.91	0.95	0.88	0.90	0.87	0.92	0.91	0.90	0.66	0.89	0.81	0.87	0.88	0.68
Cga26	0.91	0.92	0.92	0.88	0.90	0.93	0.91	0.88	0.86	0.80	0.82	0.90	0.87	0.78
Cga28	0.86	0.88	0.83	0.85	0.85	0.83	0.85	0.84	0.79	0.84	0.83	0.83	0.87	0.64
Cga32	0.79	0.79	0.64	0.72	0.85	0.87	0.85	0.85	0.81	0.77	0.85	0.87	0.79	0.64
Cga42	0.60	0.69	0.74	0.75	0.69	0.62	0.74	0.68	0.67	0.76	0.74	0.71	0.64	0.71
Cga45	0.87	0.81	0.89	0.88	0.83	0.84	0.85	0.89	0.90	0.89	0.92	0.88	0.80	0.84
Cga46	0.78	0.83	0.82	0.83	0.82	0.81	0.75	0.76	0.84	0.81	0.80	0.85	0.76	0.86

Table S2: Allelic richness at each locus for each population. Colors are attributed according to the value of allelic richness (lower values in green or white, higher values in red)

Marker	**FL13**	**FL25**	**FL26**	**FL29**	**FL31**	**FL33**	**FL35**	**FL38**	**FL5**	**FL52**	**HL12**	**HL18**	**HL19**	**HL1B**	**HL27**
Cga2	3	3	2	3	3	2	3	3	2	5	2	2	2	2	2
Cga3as	7	5	3	5	5	5	5	4	5	5	6	5	4	5	5
Cga6	3	2	3	2	3	3	3	3	3	3	3	3	3	3	4
Cga9	7	4	6	6	6	7	5	6	6	7	5	7	7	8	7
Cga11	5	4	4	4	3	7	8	6	6	5	5	8	5	7	6
Cga14	17	7	12	9	13	12	14	10	16	14	10	14	15	9	9
Cga26	14	12	13	11	14	12	11	12	12	13	11	10	11	13	13
Cga28	12	7	9	10	11	10	9	12	11	12	5	9	8	12	12
Cga32	8	7	9	12	6	9	11	9	10	9	9	9	10	9	10
Cga42	5	4	4	5	5	4	4	7	4	4	4	6	4	6	5
Cga45	10	11	11	9	8	10	9	8	11	11	8	11	10	11	12
Cga46	8	6	9	9	7	9	9	8	10	9	6	9	6	11	8

Marker	**HL28**	**HL31**	**HL4**	**HL5**	**HM13**	**HM14**	**HM19**	**HM2**	**HM20**	**HM24**	**HM3**	**HM4**	**HM5**	**HM9F**	**All pops**
Cga2	2	2	2	3	2	2	2	3	2	2	2	3	2	2	**5**
Cga3as	6	5	6	6	6	6	5	6	3	5	3	6	5	5	**9**
Cga6	3	3	3	3	3	3	3	3	3	3	3	3	2	3	**5**
Cga9	8	9	10	7	7	6	8	6	7	6	6	7	6	5	**14**
Cga11	7	7	8	9	5	6	6	5	7	5	5	7	3	3	**16**
Cga14	12	14	13	14	10	14	12	11	7	11	10	11	10	8	**32**
Cga26	14	15	13	10	13	14	13	13	10	12	10	13	9	7	**28**
Cga28	11	11	8	11	9	9	13	9	10	9	11	9	8	6	**25**
Cga32	8	6	12	10	9	10	7	9	8	7	8	9	7	5	**17**
Cga42	3	5	6	6	4	5	4	4	4	6	4	5	4	4	**10**
Cga45	11	11	11	12	8	14	9	12	12	11	13	10	8	9	**20**
Cga46	7	7	6	8	6	8	7	7	7	6	7	7	7	6	**30**
