# Supplementary material for: Gene flow and adaptive potential in a generalist ectoparasite
Source: BMC Evol Biol. 2018 Jun 19;18:99. doi: 10.1186/s12862-018-1205-2 (PMC6009953; doi:10.1186/s12862-018-1205-2)

***Additional file 2***

Probability of individual flea assignment to the k clusters computed by STRUCTURE, for each patch. The upper graph corresponds to the “optimal” number of clusters and the lower to the second highest value based on Evanno’s Delta k values. Nest labels are indicated between the two graphs. “CF” = flycatchers nests, “GT” = tit nests.

A. Fleringe

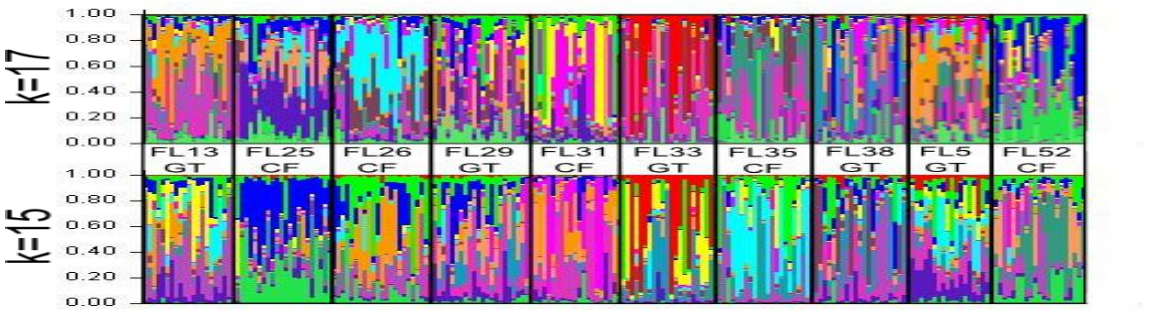


B. Hall

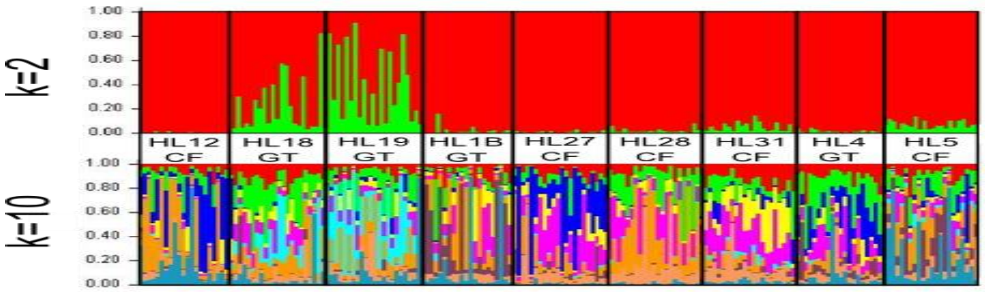


C. Hammarsänget

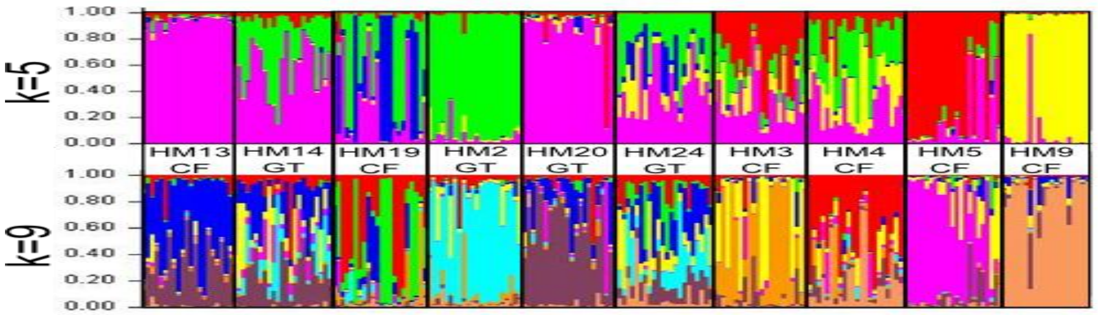

Supplement: Supplementary file 2 — This file contains figures representing the probability of individual flea assignment to the k clusters computed by STRUCTURE, for each patch (PDF 202 kb). [file 12862_2018_1205_MOESM2_ESM.pdf]
