# Supplementary material for: Gene flow and adaptive potential in a generalist ectoparasite
Source: BMC Evol Biol. 2018 Jun 19;18:99. doi: 10.1186/s12862-018-1205-2 (PMC6009953; doi:10.1186/s12862-018-1205-2)

Additional file 3

Isolation by distance within each patch, not considering host-associated structure. The solid line corresponds to a significant isolation by distance, and the dotted lines to non-significant values.

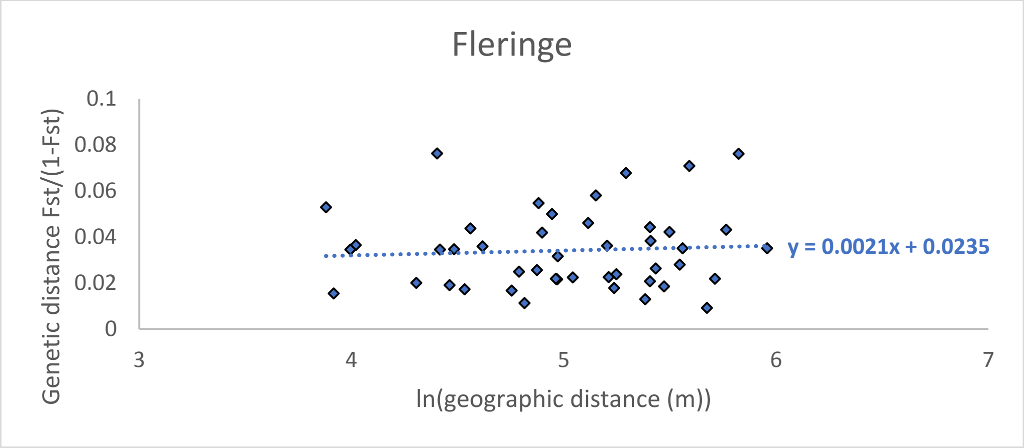

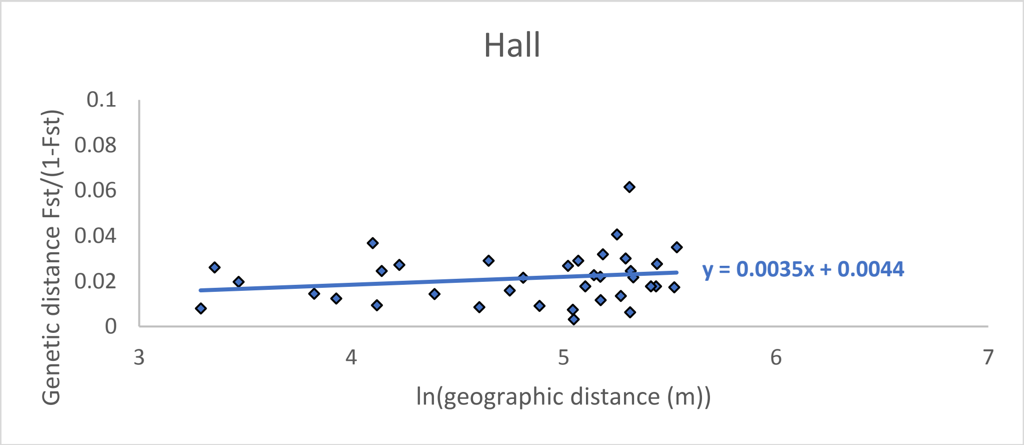

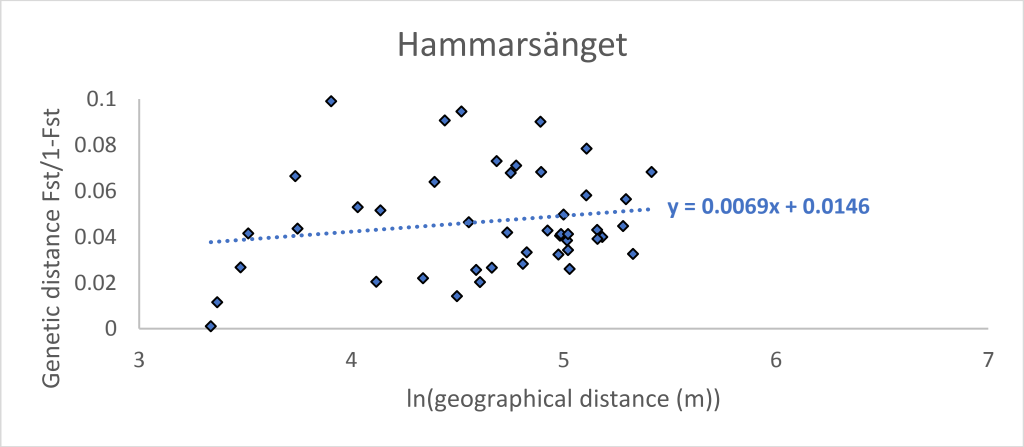

Supplement: Supplementary file 3 — This file contains graphs representing the isolation by distance within each patch, not considering host-associated structure (PDF 86 kb). [file 12862_2018_1205_MOESM3_ESM.pdf]
